# DataDesc: A framework for creating and sharing technical metadata for research software interfaces

**DOI:** 10.1016/j.patter.2024.101064

**Published:** 2024-10-01

**Authors:** Patrick Kuckertz, Jan Göpfert, Oliver Karras, David Neuroth, Julian Schönau, Rodrigo Pueblas, Stephan Ferenz, Felix Engel, Noah Pflugradt, Jann M. Weinand, Astrid Nieße, Sören Auer, Detlef Stolten

**Affiliations:** 1 Forschungszentrum Jülich GmbH, Institute of Climate and Energy Systems (ICE) – Jülich Systems Analysis (ICE-2), 52425 Jülich, Germany; 2 TIB - Leibniz Information Centre for Science and Technology, 30167 Hanover, Germany; 3 Department for Computer Science, Carl von Ossietzky University of Oldenburg, 26129 Oldenburg, Germany; 4 L3S Research Center, University of Hannover, 30167 Hannover, Germany; 5 RWTH Aachen University, Chair for Fuel Cells, Faculty of Mechanical Engineering, 52062 Aachen, Germany

**Keywords:** research data management, RDM, FAIR, software metadata, metadata schema, interface description, semantic software description, software publication, software reuse, machine actionable, software documentation

## Abstract

The reuse of research software is central to research efficiency and academic exchange. The application of software enables researchers to reproduce, validate, and expand upon study findings. The analysis of open-source code aids in the comprehension, comparison, and integration of approaches. Often, however, no further use occurs because relevant software cannot be found or is incompatible with existing research processes. This results in repetitive software development, which impedes the advancement of individual researchers and entire research communities. In this article, the DataDesc (Data Description) framework is presented—an approach to describing data models of software interfaces with machine-actionable metadata. In addition to a specialized metadata schema, an exchange format and support tools for easy collection and the automated publishing of software documentation are introduced. This approach practically increases the FAIRness, i.e., findability, accessibility, interoperability, and reusability, of research software as well as effectively promotes its impact on research.

## Highlights

• The DataDesc framework focuses on research software interfaces and their data models
• A metadata schema maps input and output content, formats, value ranges, and structures
• Tools enable the collection, exchange, and publication of machine-actionable metadata
• DataDesc reduces annotation efforts and promotes software reuse and integration

## The bigger picture

Combining and integrating software and data is critical to answering complex questions in many areas of research, but identifying compatible software is a time-consuming task. Frameworks, such as DataDesc, that facilitate the discovery and integration of software could promote reuse and scientific exchange between research groups, thus reducing resources wasted by redundant software development.

## Teaser

The authors emphasize the importance of research software but point out that it is often difficult to reuse and integrate within individual software workflows. To facilitate this, they advocate specifically documenting software interfaces and their data models as machine-actionable metadata. For this, they present the DataDesc metadata schema and support it with tools for collecting, exchanging, and publishing the information. The authors show how the reusability and interoperability of research software can be increased while minimizing annotation efforts.

## Introduction

### Motivation

Research in many academic disciplines relies on computational methods to the degree that the utilization of software has become integral in numerous fields. Thus, the efficient discovery and reuse of research software is essential for academic progress and communication.^1^ Furthermore, the examination of open-source code aids in the comprehension, comparison, and integration of methodologies, and the application of software enables users with various academic backgrounds to replicate, validate, and build upon study findings. Research software publications are also becoming increasingly important for measuring the research impact and so for the reputation of individual researchers.^2^^,^^3^ Furthermore, workflows have become a popular means in various domains to facilitate the execution and reusability of complex, multi-step computational processes.^4^ Machine-actionable specification of data and software interfaces would allow for the suggestion of suitable tools and data during workflow design, as well as validating inputs and outputs during execution.^4^

However, finding compatible software that meets researchers’ content requirements and integrates seamlessly into existing research workflows remains a significant challenge.^5^ Currently, available research software metadata schemas, such as CodeMeta,^6^ only focus on general information and omit detailed technical descriptions of interfaces, which are important for interoperability and subsequent use.^7^ At most, such information can be found on software documentation sites, where it is neither standardized nor machine actionable. Furthermore, metadata are stored and exchanged in various formats, from which no standardized exchange format has yet been developed that would allow the broad reuse of metadata once they have been captured.^8^ Therefore, in order to make a software known on various platforms and increase its impact, metadata must often be repeatedly collected for each platform separately, which greatly increases documentation effort, which is already perceived to be high. At the same time, the broad dissemination of metadata is essential for the long-term discoverability and subsequent use of software.^9^ As a result, researchers must invest considerable effort in both documenting and publishing metadata, as well as finding and integrating research software. Every time software is not found and reused but instead redundantly developed, a significant increase in avoidable programming, documentation, and maintenance efforts is imposed.

To address these issues, adaptations of the FAIR guiding principles, which aim to increase the findability, accessibility, interoperability, and reusability of research data,^10^ were recently adopted specifically for research software.^11^^,^^12^ Among other things, these principles require research software to be registered and indexed in searchable platforms and annotated with rich metadata. In order to increase the interoperability of software components, the metadata must include interface definitions of modular software architectures, making interoperability the most challenging among the four high-level principles. On the one hand, all metadata must comply with domain-relevant community standards in order to be easily understandable for researchers. On the other hand, the metadata must be machine actionable for automated software discovery. In practice, however, it is unclear how the postulated abstract principles may be put into action.^13^

The Data Description (DataDesc) framework presented in this article is a practical approach to improving the interoperability and findability of research software.^14^^,^^15^ It centers around a software metadata schema that describes the data models on which software interfaces are based. In order to capture characteristics that are usually only described in the documentation, metadata elements from established schemas were reused, combined, and supplemented with new ones. In addition, the framework provides an exchange format in which this information is mapped in a machine-actionable manner. The hierarchical data structure of the OpenAPI standard was chosen as its basis to facilitate its reuse in automated processes. Finally, it includes a toolset that makes it easy to capture and publish software metadata from the source code.

The remainder of this article is structured as follows: the related work section presents a review of existing software description schemas, addressing different formats in the tension between metadata and documentation. Furthermore, automated description tools and software publication platforms are compared on the basis of the metadata formats they generate or use. The results section explains the different components of the DataDesc framework. First, the DataDesc schema is described, along with the typical data flow between individual interface components of research software on the basis of its contents, formats, value ranges, and structures. Then, an explanation of the structure of the exchange format and the individual tools that support metadata generation is given. Finally, pipelines to publication platforms are described, with which the metadata can be disseminated in a partially automated way. Completing the results section, the presented approach is exemplarily applied to the Framework for Integrated Energy System Assessment of the Energy Transformation Pathway Optimization Suite (ETHOS.FINE)^16^^,^^17^ from the energy domain. The article concludes with a discussion section highlighting the key characteristics of the presented approach as well as its limitations and provides an outlook on future work.

### Related work

This section provides an overview of current software description schemas. Additionally, automated description tools and software publishing platforms are contrasted.

#### Software description schemas

##### Software metadata standards

A metadata schema defines a set of metadata properties to standardize the structure and semantics of descriptions of artifacts within its scope.^18^ A metadata standard may further standardize the encoding format, what values are allowed for which properties, and how they should be represented (e.g., conventions for spelling, capitalization, date formats), among other things.^18^

Many different metadata schemas and standards exist for a variety of use cases. Whereas Dublin Core^19^ outlines general metadata terms, the DataCite Schema^20^ focuses on describing research data, and the Data Catalog Vocabulary (DCAT)^21^ focuses on interoperability between data catalogs. Schema.org is intended to describe web pages with structured data markups, but it is also widely used for other purposes.^22^

With respect to research software, CodeMeta is a popular community-driven metadata standard. It is based on schema.org, which it augments with several additional terms. Various crosswalks exist—that is, mappings from one schema to another—between CodeMeta and other metadata schemas. CodeMeta covers many aspects of software metadata, with some terms focusing on technical details, such as file size or operating system, and others on administrative information, like licenses and links to the software repository. It supports the unambiguous assignment of authors, contributors, licenses, and more via uniform resource identifiers (URIs). The purpose of a software can be specified by means of a textual description, application categories, keywords, and a link to a README file or reference publication. Apart from a coarse classification, the declaration of a software’s purpose is therefore still far from being readily machine actionable, that is, without interpreting (or misinterpreting) natural language. Furthermore, CodeMeta does not include terms for specifying the input and output of a software, nor does it include terms for specifying features or methods implemented by a software. Similarly, the Citation File Format schema defines general metadata for the citation of software repositories without describing the software’s purpose and interface.^23^

In the domain of geoscience, Garijo et al. developed the Software Description Ontology^24^ by extending their own approach, namely OntoSoft.^25^ OntoSoft properties are structured in six categories: identify, understand, execute, do research, get support, and update. The ontology captures technical metadata like programming language and dependencies and descriptive data like name, website, and contributors. The authors added a description of the input and output data also utilizing the Scientific Variables Ontology and aligned OntoSoft with CodeMeta. The metadata are published to an open knowledge graph.^26^ Garijo et al. support the linking to other instances in the semantic web, like Wikidata, the Scientific Variables Ontology, and others. Additionally, they developed programs to support researchers in metadata creation and the search for software models.^27^^,^^28^

In the domain of bioinformatics, Ison et al. developed the metadata standard biotoolsXSD for the software registry bio.tools.^29^^,^^30^^,^^31^ The metadata is expressed as an XML schema and contains 55 properties, 10 of which are mandatory. The use of the EDAM ontology as value vocabulary is required for properties such as function, input, and output. The metadata schema also contains software-specific properties like programming language, license, and operating system. The use of an ontology is not required for these.

##### Interface description standards

In order to increase its technical interoperability and reusability, software can be documented by means of interface description languages. The syntax of such a language enables the formal and programming language-agnostic description of interface functions and their parameters. Well-known representatives include the Web Service Description Language (WSDL)^32^ and Web Application Description Language (WADL).^33^ Both are XML-based specification standards that describe the syntactical elements of web services and, primarily, how to access them. They are utilized to simplify the information exchange in Web 2.0 application development. Whereas WSDL is used in conjunction with the Simple Object Access Protocol (SOAP), WADL enables the description of web services conform to the Hypertext Transfer Protocol (HTTP) and in particular the Representational State Transfer (REST) paradigm. Both languages provide machine-processable descriptions but do not support taxonomy or ontology information for semantic classification. The WSDL and WADL standards were last updated in 2007 and 2009, respectively.

The OpenAPI Specification is an interface description language that focuses on REST APIs.^34^ By utilizing YAML and JSON, it is both machine actionable and human readable. By default, it is used to define the general properties of APIs, such as the version, contact, and license information or server names and addresses. However, it also defines technical aspects, mainly with respect to REST interface functions like the paths to endpoints, HTTP verbs, parameters, or response code descriptions. The OpenAPI standard also allows for the annotation of custom properties using a concept called extensions or x-attributes. These extensions provide a powerful way of describing additional functionality not covered by the standard specification. As an open and non-proprietary state-of-the-art industry standard, the OpenAPI Specification is actively maintained and regularly updated.

The Web Ontology Language for Web Services (OWL-S) defines ontologies built on top of the OWL for describing semantic web services on a technical level, making it more powerful but also more complex than regular description languages (*inter alia* [i.a.], WSDL and WADL).^35^ It describes the purpose of services, how they are accessed, and how they function. Although more powerful than comparable description languages, OWL-S is not an “end-all-be-all” solution to service descriptions and requires domain-specific ontologies for describing domain-specific functionality. Furthermore, its focus on semantic web services greatly reduces its legibility; it was last updated in 2004 and is not suited to easy human reading.

The Functional Mock-up Interface (FMI) is an open-source standard for simulation software interfaces.^36^ All simulation models whose interfaces have been designed along the standard become so-called functional mock-up units (FMUs). The standard ensures that all FMUs are compatible with one another and can be executed in combination on the basis of XML and binary files and C-code, which defines functions, variables, and mathematical formulas. The FMI comes with its own documentation standard, namely the FMI Description Schema, which only applies to FMI conform software. It encompasses general information regarding the FMUs, such as name, version, author, and license, as well as technical information like model structures, unit, and type definitions. The schema allows structured extensions to the base standard in order to flexibly meet additional requirements. The FMI is still actively maintained today and is used in many industrial companies.

##### Non-standardized software description

Software metadata are also described on web pages, where the use of specific terms is typically enforced but without adopting a metadata schema, thereby only establishing uniformity on the web page itself. Schwarz and Lehnhoff, for example, describe a catalog of energy co-simulation components.^37^ They use a semantic media wiki to collect information on simulators and add descriptions to the simulation interfaces. The elements of the catalog, which can be used for a metadata schema, are not described in greater detail. The open energy modeling initiative (openmod) includes a list of energy models in their wiki.^38^ For each of these, administrative and descriptive metadata are listed, such as license, link to a code repository, and model class. The descriptive elements include detailed information on the models. The elements are not formalized as metadata schema, and controlled vocabularies are used for neither the elements nor the values. The Open Energy Platform (OEP) introduces framework and model factsheets to describe frameworks and models.^39^ These descriptions have been further developed based on the non-formalized openmod metadata elements.

In addition to the websites that focus on the general description of software using metadata, there are documentation and specification websites that focus on describing the source code of the software, providing detailed technical guidance for both users and developers (e.g., see the PyPSA documentation^40^). The design ideas and specific technical elements of software are typically defined along with their underlying algorithms and procedures. Specifications for the API, user manuals, and examples of applications make it possible to correctly utilize the software. Software documentation is predominantly written in natural language and, therefore, is neither machine actionable nor easily searchable or comparable. Although such documentation provides rich information, it is not typically considered to be part of software metadata.

It should be noted that existing software metadata schemas do not include technical documentation about interfaces. Although interface description languages are designed to collect this information, they focus on web services and protocols. As a result, the interface information of software that is not provided as a service is primarily published as non-standardized and non-machine-actionable information on web pages, often without any connection to controlled vocabularies or ontologies. For research software, most of which is not provided as a service, there is not yet a suitable schema that enables semantic interface descriptions. However, the code-near structures of interface description languages and the ability to connect some via extensions to established software metadata schemas offer promising foundations.

#### Software description tools

Documentation is generally regarded as an essential component of software development, and yet it is frequently neglected. This is often due to the fact that considerable effort is involved in writing detailed, well-structured, and version-controlled documentation. A recommended means of alleviating this issue is the use of automated documentation tools,^41^ which are specifically designed to aid in the process of creating comprehensible and complete documentation for a software project. There are many such tools available, and although the general objective is the same, they differ in their approach, programming language, or input and output formats.

Many of these tools, e.g., Javadoc^42^ or Perldoc,^43^ focus on single programming languages and use source code as their main inputs. By parsing the code, they obtain information on defined types and functions and their relationships. Some documentation tools, such as Doxygen^44^ or the Sphinx plugin Napoleon,^45^ are able to extract this kind of information from bare code; other tools, however, rely on code comments in a determinate format. In either case, additional metadata are typically conveyed via comments. This can comprise, for example, a general description or explanation of a function’s parameters. Such information is mostly given as free text and is placed into the final documentation without change. MkDocs^46^ and, in some cases, Sphinx^47^ constitute an exception by only manually parsing created files, e.g., containing reStructuredText. They can, however, both be extended with plugins that automatically generate said text files from code. The output of documentation tools is nicely formatted documentation pages, typically using HTML or LaTeX. These pages are easily readable and comprehensible to humans but hardly machine actionable. Roxygen2^48^ also generates intermediate files that are, in theory, machine actionable but, due to their custom data format, are limited in their reusability.

In this regard, Swagger^49^ can be distinguished from other tools. Swagger is used primarily for documenting REST APIs and provides a set of distinct but related tools for that. At its core, Swagger utilizes a YAML or JSON file standardized in the OpenAPI Specification. This file is machine actionable and stores all metadata of an API in a structured, hierarchical way. It can be created manually or generated from code and, when passed to the appropriate Swagger tools, is used to generate a human-readable documentation web page. Unlike many other tools, Swagger does not require specially formatted comments within the code in order to extract the information. Furthermore, Sphinx can be extended by a plugin to enable support for OpenAPI Specification files, which, as implied, makes it possible for Sphinx to generate interface descriptions from OpenAPI-compliant YAML or JSON.

It should be highlighted that software and, therefore, interface documentation can be parsed automatically from source code, and many documentation tools are available. However, most of these tools rely on code comments that are formulated in natural language and, therefore, are not directly machine actionable. In this regard, Swagger is an exception, as it centers around a universal, machine-actionable, and standardized metadata file, which is suitable for documentation pages as well as automated reuse. Even though Swagger is intended only for documenting REST interfaces, there is no lock-in to individual programming languages. Because of this inherent flexibility, it offers some potential for the development of generic software documentation workflows.

#### Software publication platforms

Software can be made discoverable and available for reuse by being published on a variety of software-specialized publication platforms. Therefore, the distinct purposes and objectives of these platforms vary. Although some store the source code of a software in versioned repositories (e.g., GitHub^50^), in particular, to enable its further development, others aim at the distribution and easy integration of mature programs (e.g., Anaconda^51^). Some platforms serve as registries, indexing large collections of software and making them searchable using detailed metadata (e.g., Python Package Index [PyPi]^52^). Others are dedicated to the provision of technical documentation and user guides (e.g., ReadTheDocs^53^).

Furthermore, most of the software publication platforms differ in the data formats they accept and the uploading processes they provide. Even when using similar file formats, the required information or information structures vary. Some platforms, such as GitHub,^50^ GitLab,^54^ Bitbucket,^55^ or SourceForge,^56^ ingest the source code directly without a specific required structure. Others support the inclusion of metadata configuration files. For example, Anaconda Distribution^51^ requires a YAML file that describes the project. Maven Central^57^ requires an XML POM file for storing metadata. Whereas PyPi^52^ requires a TOML file with information about packages, NPM^58^ generates a JSON file based on text prompts. SwaggerHub^59^ requires an OpenAPI-conforming interface description file in YAML or JSON formats, containing function and argument specifications. Like ReadTheDocs,^53^ some platforms require a software project to have a documentation folder according to a standard. In this specific case, Sphinx or MkDocs can be used in order to generate such a folder. Platforms like GitBook,^60^ CRAN,^61^ or GitHub Pages^62^ require programming language-specific files for the installation. For example, submitting a project to CRAN requires first creating a TAR.GZ file. GitHub Pages^62^ can store project documentation via HTML files. The OEP,^39^ Open Research Knowledge Graph (ORKG),^63^^,^^64^^,^^65^ or bio.tools,^30^ for example, require manually filling forms with metadata in order to register it.

There is no question that publishing platforms are critical to the dissemination, findability, and reusability of research software within and across academic communities. It is advantageous to employ various platforms in parallel to utilize their distinct strengths to increase the impact and transparency of a software. However, as no uniform format for the exchange and subsequent use of software metadata has yet been identified, metadata must often be collected redundantly and adapted to heterogeneous formats and processes, creating the need for a machine-actionable and programming-language-agnostic exchange standard.

## Results

This section introduces the DataDesc framework. As a central component, the DataDesc schema, which enables the thorough description of software interfaces, is explained. Then, an exchange format and assistance tools are presented, enabling the gathering, storage, and reuse of machine-actionable metadata. Thereafter, procedures that can be used to share metadata on publishing platforms are defined. Finally, the annotation of a research software according to the DataDesc schema is demonstrated. DataDesc has been released with all of its components presented here under the open MIT license on GitHub.^14^ In addition, the current version 1.0 has been made available in the JülichDATA repository under the CC0 public domain dedication.^15^

### DataDesc schema

Metadata schemas often focus on general information provision, which primarily includes the naming of organizations and persons involved in the development process and the technical and licensing conditions under which the software can be obtained and used. By specifying categories and keywords, they also make a valuable contribution to supporting the findability of software. Within these schemas, however, the description of interfaces can only be superficially embedded in general metadata properties. Although this information already provides important insights into a software, it is not sufficient to facilitate its interoperability and reusability in a machine-actionable way.

To compensate for this omission, the DataDesc metadata schema for the description of research software was developed. In addition to capturing general information relevant to the scientific context, it aims, in particular, at the detailed documentation of software interfaces. The programming language agnostic schema offers the possibility to treat all functions of an interface and its input and output parameters individually. The structured capture of information, which is often only available in this level of detail in the form of instructions and specifications written in free text, enables their automated processing and increases their findability and comparability. Mapping them as machine-actionable metadata allows both humans and computers to discover and understand the capabilities of a software, interact with it, and integrate it with other programs and data without having to refer to the source code or further documentation.

The structure and content of the DataDesc schema are based on the OpenAPI Specification, which is a widely used language for the standardized description of web interfaces based on HTTP and REST. However, since research software mostly does not follow a client-server architecture and is not provided as services accessible via web interfaces, DataDesc does not focus on the transmission of data via HTTP requests to be processed on the server side but on the permissible use of locally installed programs. Largely adopted from OpenAPI are the hierarchically organized documentation structures, which allow code-near descriptions of interface elements. Furthermore, the data type specifications based on JSON schema are used, with which even nested data models used by an interface can be mapped in detail. In the area of the general description of research software, the CodeMeta metadata standard and the Schema.org ontology on which it is based were closely followed, and adopted metadata properties were integrated within the hierarchical documentation structure. As a result, the DataDesc schema is largely compatible with both the OpenAPI and the Schema.org or CodeMeta standards and minimizes the amount of necessarily individually defined terminology.

**Figure 1:**
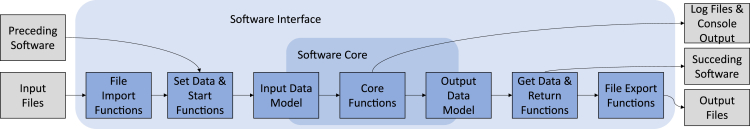
Schematic representation of the generic information flow between software interface and core components

**Figure 2:**
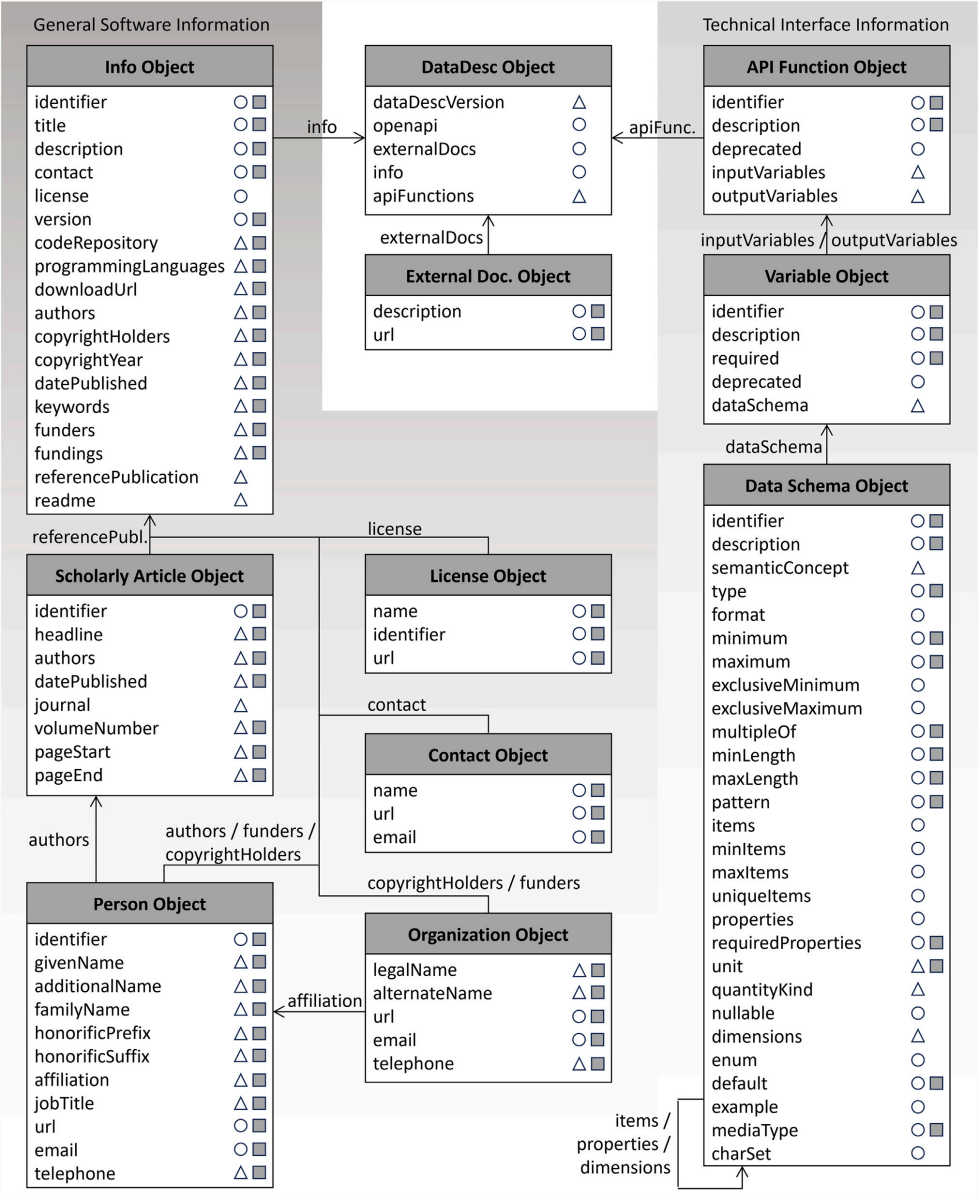
Structure and content of the DataDesc schema for describing software and their interface data models Both the general and technical information are organized in information objects, with arrows indicating the different relationships between them. DataDesc properties that comply with the OpenAPI Specification directly or via extensions are marked with white circles or white triangles, respectively. Properties that map to the Schema.org ontology are indicated by a gray square. Individual property definitions can be viewed at GitHub: https://github.com/FZJ-IEK3-VSA/DataDesc/blob/main/schema/DataDesc_schema_v1.1.md.^14^

An interface, as schematically depicted in Figure 1, serves as a connection point for users and programs to interact with a software. It is composed of the functions through which data can be inputted into and retrieved from the software. These functions are distinguished from the inner functions, which form the logic of the software core. The program core can only be addressed indirectly via the interface, whereby the structures and formats of the information flow are defined by the interface functions and internal data models. An interface description performed with the DataDesc schema describes a software in general and formally identifies the characteristics of an interface according to a collection of metadata elements. The meaning and use of the interface property elements, detailed in Figure 2, is described in the following. References to schema elements are in italics and parentheses.

The schema comprises the naming (identifier) and description (description) of all functions, which are part of an interface and over which a software can be addressed (apiFunctions). In order to enable easy and, in particular, error-free use of a software, the functions’ parameters, as well as their underlying data models, must be described in detail. To adequately characterize variables serving as the input or output parameters of interface functions (inputVariables, outputVariables), their intended and allowed data must be described in terms of contents, formats, values, and structures.

#### Data content description

In order to digitally process information, it must be stored in the form of variables. In the course of software development, the data content of each variable is defined. This refers explicitly to the referencing of real-world concepts, such as the height or weight of a person, and not of data types, which specify whether variables can contain integers, floats, strings, or similar. A precise understanding of the meaning of the data content a software requires, processes, and outputs is essential for its correct and intended use.

However, capturing meanings is not a trivial task. Depending on the demand for precision and generality, describing data content involves varying degrees of effort. The easiest approach is to sensibly name variables during software development (identifier) and explain them further in docstrings (description). However, these names and free-text descriptions almost always leave considerable room for interpretation as to the meaning of the data content. Instead, it is more interoperable to reference concepts from ontologies with their respective URIs (semanticConcept), which often provide unambiguous definitions that are agreed upon in the respective research domain.^66^ Of course, the open collaborative development of such concepts with the broadest possible participation and agreement within a domain is a labor-intensive process requiring well-organized community infrastructures.^67^

If the variable is numerical, then documenting the meaning alone is insufficient for fully describing its data content. In this case, additional information about a unit is necessary, so that, for example, a duration of 7 h can be distinguished from one of 7 s. Just as with the concepts before, a unit can be specified by an identifier or an ontology reference (unit).

In the context of software interfaces, specific concepts and units of measurement need not always be declared. In order to enable a greater degree of freedom in data entry and so to enable a more flexible application of a software, intended data contents can be more broadly indicated. For example, specifying the general concept means of transport indicates that the software can process data about bicycles, trains, cars, and so forth. Likewise, a generic quantity kind such as length can be indicated for an interface parameter that expects, for example, a height, width, or distance value so that a specific unit of length such as meters, centimeters, or miles can be selected when entering data (quantityKind). In this context, the use of ontologies offers the advantage that they often already include information that specifically relates to more general concepts.

#### Data format description

The format of a variable defines how the information it contains is to be encoded into binary data and subsequently interpreted. It provides information about which operations may be applied to the data content. The format is defined by the data type of a variable (type). There are primitive and complex data types that can be native to programming languages or that come as custom data types provided by libraries. Primitive data types, such as strings, integers, or booleans, can hold single values. Complex data types, like lists, tables, arrays, or classes, can group multiple instances of primitive data types. The data type of a variable can be further specified by hinting at, e.g., specific numeric types, string formats, or object types (format).

As complex data types can also recursively contain complex data types, nested structures of arbitrary depth and complexity are possible, although their final level can only contain primitives. Complex structures of this kind are often used to define data models, which summarize the input and output data of research software into single data objects and make them centrally accessible (e.g., ETHOS.FINE data model,^68^ IAMC pyam data model^69^). Oftentimes, classes are used at the highest level for the representation of such data models, to which the interface functions for importing and storing, as well as for reading out and exporting, are assigned (cf. Figure 1). In order to describe not only the data types of the function parameters but also the data types nested within them, hierarchical data formats are mapped in the DataDesc schema using recursive relationships, through which the data schema of a variable can contain further data schemas (cf. Figure 2).

Files represent another complex data type, as they can also contain and group data of arbitrary types. As is shown in Figure 1, reading in files is a widely used method of transferring data to a research software, which is why an interface description must also inform regarding permitted file formats that can be processed without errors. For each variable containing a file reference, whatever the variable itself, e.g., string or file object, DataDesc gives the option of specifying the format (mediaType), e.g., text formats like XML, HTML, or TEXT or binary formats like PDF, XLS, or JPG, of a referenced file. Beyond that, the character encoding (*charSet*) of text formats, e.g., UTF-8, ASCII, or ISO 8859-1, can be specified to support the correct interpretation of text data.

#### Data value description

When creating software, a permissible value range must be defined for each variable, guaranteeing the technically error-free processing and consistency of content for all values from within this range. Technically, the value range is defined in many programming languages by the choice of a variable type. In Java, for example, a variable of type float allows all floating-point values from $-3.4\times 10^{38}$ to $3.4\times 10^{38}$, whereas a boolean can only accept the values of true and false.

In addition, a value range can be further limited based on content considerations. For example, if a longitude is to be stored in a float variable, only floating point values from −180 to +180 degrees should be considered valid (minimum, maximum, exclusiveMinimum, and exclusiveMaximum). It is also possible to specify that a number must be the multiple of another number (multipleOf). For text variables, the number of characters can be limited (minLength, maxLength). If they are only allowed to contain certain patterns, this can be defined through regular expressions (pattern). For example, if a filename is to consist of only letters, numbers, and underscores, this can be defined using the expression ˆ[A-Za-z0-9_]+$. If the allowed value range of a variable should be fully restricted to predefined values, e.g., North, East, South, and West, DataDesc schema offers the possibility of documenting them in the form of value enumerations (enum). Regardless of the variable type, it is important to document whether null values can be processed without errors (nullable).

It is also part of the value description to specify whether a variable is an optional parameter (required). If this is the case, then it does not need to be set when the respective function is called upon. In this context, a default value can also be specified and is used if the variable is not explicitly set (default). For complex data types, it also can be specified which property variables are mandatory (requiredProperties) and whether contents may only occur once (uniqueItems). Finally, examples can be specified in the DataDesc schema for a better understanding of the data values (example).

#### Data structure description

For variables of complex data types, the internal data structures must be described at both a technical and contextual level so as to enable the correct accessing of individual values and the determination of their respective meanings. The processing procedures of software programs are designed on the basis of specific data structures whose declaration is the task of interface descriptions. The correct function of a software is not ensured if the structure of the passed data differs from the expected data structure.

**Figure 3:**
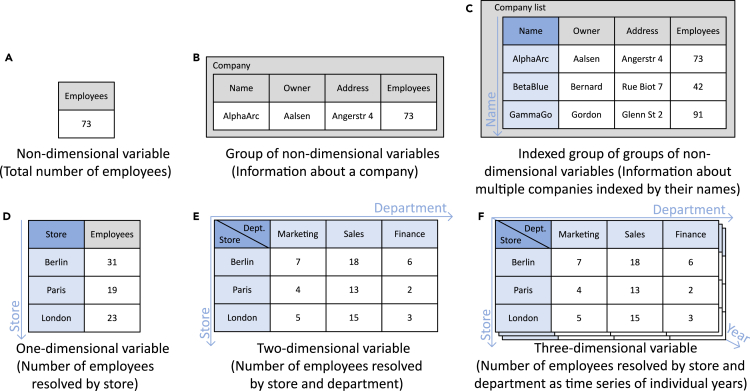
Comparison of widely used data structures based on an example of information about companies Variables are shown in gray and their values in white, whereas dimensions are displayed in dark blue with their indices in light blue.

Figure 3B shows four independent variables of the primitive data types of integer and string, which, as they represent information characterizing the same single object, are combined in a grouping variable company, which itself must be described. The technical structuring of the data thereby maps its context by relating the four variables to each other and to the grouping variable: number of employees becomes number of employees of the company AlphaArc. In order to capture this kind of context within an interface description, the DataDesc schema utilizes the hierarchical structure to map relationships between variables. For example, an object such as company is described using a collection of property-value pairs (properties), whereby a data schema object must be specified for each property variable, in this case for name, owner, address, and employees.

In addition to grouping, the dimensional resolution of information represents a significant structural pattern. Figures 3A, 3D, and 3E show the increasing resolution of the initially non-dimensionally resolved information: the AlphaArc company has a total of 73 employees. This information does not change subsequently, but the single value is broken down into individual values per store and then per store and department. Each dimension along which the information is resolved is listed as part of the variable (dimensions). Each dimension is mapped as a child object and can be described in more detail, e.g., with regard to meaning and allowed index range. In this context, the combination of dimension indexes is unique, which is why it acts as a key and enables the unique identification and retrieval of each individual value. At the same time, an individual context is defined for each value: 15 employees is the team size of the sales department in the London store, for example.

The structural description provides not only information about the context of values but also about data access mechanisms that might be expected by interface functions (see Figure 3C). The structure of the company list, which can be, e.g., in the format of a Python dictionary, pandas DataFrame, Java HashMap, or SQL table, contains several uniformly designed datasets (items). The variable name was determined as a key index due to the identifying character of its values. As a dimension of the company list, the variable name allows access to individual datasets. For a grouping variable, the number of items that it should list can be limited (minItems, maxItems).

Figure 3F shows another structure that combines grouping and resolution by adding the third dimension *year* to the resolution of the number of employees based on the two dimensions of store and department. Here, the total number of 73 employees is not broken down further but put in the context of a specific year, e.g., 15 employees is the team size of the sales department in the London store in the year 2010. Together with uniform information for, e.g., the years 2015 and 2020, this third dimension turns the dataset into a time series.

The DataDesc schema avoids redundant descriptions of complex data models that are shared by multiple function parameters. Variables and their data schemas are described separately. In that way, a data schema object can be referred to by multiple variable objects (cf. Figure 2).

### DataDesc exchange format and utilities

In addition to the schema for describing software and software interfaces, the DataDesc document, an exchange format for the integrated storage and flexible subsequent use of software metadata, represents the second core component of the DataDesc framework. The OpenAPI Specification was chosen as its foundation, as it allows a programming-language-agnostic description of software that is usable by both humans and computers. A software is described in a single JSON file. The basic structure of a DataDesc document consists of a hierarchical object tree that is subdivided into the two sections general software information and technical interface information (compare Figure 2). In the general software information section, all general information is accommodated. If metadata elements are required for this that are not provided for in the OpenAPI Specification, then they can be added by means of x-attribute extensions without violating the standard. In the technical interface information section, the technical interface metadata, as described by the DataDesc schema, is specified. The x-attributes again provide the opportunity to compensate for missing OpenAPI metadata elements. In addition, they form the basis for using the standard not only to describe REST-compliant interfaces; they can also be used to arrange and annotate code elements such as classes, functions, and parameters in the hierarchical object tree according to individual software interface designs.

In order to support the description of software based on its general properties (such as author, license, programming language, funder, etc.) and the transfer of this information into the exchange format chosen in the DataDesc approach, a browser-based input form was added to the framework. The metadata fields of the form thereby widely map to the Schema.org ontology, as this is already widely used, e.g., by the CodeMeta standard, and can be applied across research domains.

Unlike the general metadata, the technical documentation is produced directly in the source code of a program, which is why the definition of a machine-actionable formatting of this information, as well as its automated parsing and transfer into the exchange format, must be made individually for each programming language. In the context of Python software, code components related to the interface are individually marked up with DataDesc schema elements by utilizing the Python library typing.Annotated.^70^ A Python parser specifically developed for this schema extracts both the relevant code structures and their metadata and automatically generates the hierarchical object tree from them, which is then stored in an exchange-format-compliant file.

The DataDesc utilities are complemented by a tool for merging the metadata files so that the entire description of a software can be represented in a single concise DataDesc document that is easily exchangeable. Its OpenAPI conformity also ensures high interoperability, as a multitude of publicly available tools can be applied to it.^49^^,^^71^

### DataDesc publication pipelines

Making it possible for developers to create software metadata and documentation only once and then flexibly reuse it is one of the main objectives of the DataDesc approach. Against this background, technical processes are defined and, where necessary, supported with scripts that enable the collected information to be disseminated on software publication platforms. These publication pipelines are unique to each platform and subject to automation. To upload data to any of the publication platforms mentioned below, a free user account must first be created.

The DataDesc document can be uploaded and published on SwaggerHub^59^ via its GUI or API, without any need for modifications. To publish the description on GitHub,^50^ it is sufficient to add the file to the software’s versioned repository and reference it in the central README file. To make the documentation more visually appealing, a link to a SwaggerHub-hosted documentation page can be included. The registration of Python-based software and its metadata in the PyPi^52^ has been fully automated. With a DataDesc script utilizing restructuring and conversion tools, the JSON file and corresponding software source code are reformatted, uploaded, and published on the platform. The publication on ReadTheDocs^53^ can also ingest information based on a DataDesc exchange file. In order to upload the documentation in the appropriate format, it must first be created using, for example, Sphinx with its extension for the parsing of OpenAPI Specifications. Then, a GitHub repository comprising the generated documentation can be imported. The ORKG^63^^,^^64^^,^^65^ provides a GUI and an API for uploading software metadata, which can be entered manually into a form. In addition, a script was added to the DataDesc framework to automate the translation of the exchange format into the ORKG template structures.^72^ Currently, this mapping must still be performed individually by each user. However, work is underway to include this functionality in the ORKG. As part of the development of the Open Energy Knowledge Graph,^73^ efforts are being made to ensure that the exchange format can also be processed directly within the OEP.^39^

### Application case

**Figure 4:**
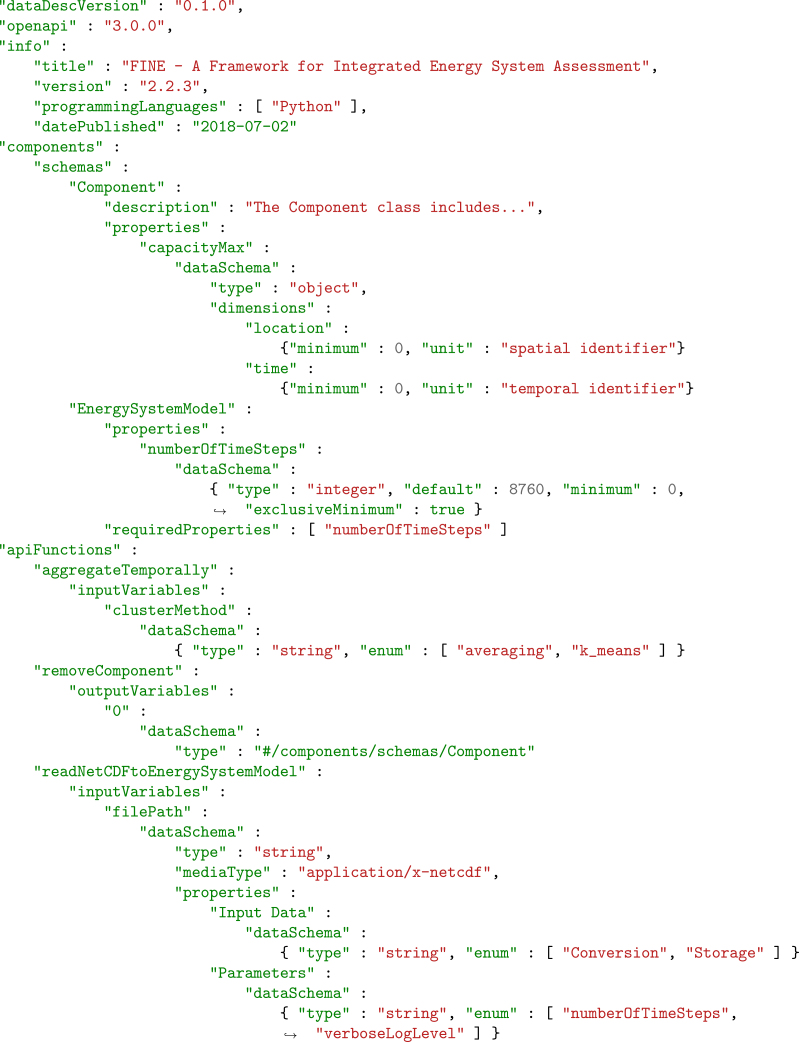
Compilation of selected lines of code from the DataDesc document generated to describe the ETHOS.FINE interfaces to represent the syntax and its semantics within the DataDesc schema Most brackets have been omitted to preserve brevity.

To demonstrate the application of the DataDesc approach, it is applied to a research software in the following. For this, the open-source ETHOS.FINE framework^16^^,^^17^ was chosen to illustrate a use case that is both realistic in terms of complexity and intriguing with respect to the interfaces provided. Using selected excerpts from the created interface documentation shown in Figure 4, the syntax of the JSON file generated using the DataDesc utilities is presented, and the semantic expression capabilities of the DataDesc schema are assessed. To allow for a more in-depth review of the entire DataDesc approach, all code and documentation files created as part of this example application are published in the DataDesc repository.^14^^,^^15^

ETHOS.FINE is a Python package with a 5-year development history originating in the research domain of energy systems analysis.^17^ It enables the modeling, optimization, and evaluation of energy systems. In addition to accounting for technical and environmental constraints, its optimization also seeks to minimize total annual energy system costs. It supports the creation and computation of spatially, temporally, and technologically highly resolved models while integrating complexity reduction techniques to shorten computation times. In its current version, v.2.3.1 from 2023, the framework includes around 20,000 lines of code (excluding blank lines) and 10,000 lines of code documentation. Whereas the source code of the software project is hosted on GitHub,^74^ the user documentation is published on ReadTheDocs.^75^ The documentation pages are based on the inline docstrings in the source code and were automatically generated using the Sphinx package. In addition, a short entry in the OEP’s software framework list was written for the framework.^76^

The ETHOS.FINE software is based on a central data model, namely the Energy System Model (ESM), which is represented by the ESM and component classes. It holds multi-dimensional information pertaining to the energy system under investigation and comprises all characteristics of its components, e.g., for the provision, transmission, and storage of energy. As input, besides basic parameters for calculation control, the software requires the general conditions of the energy system and the techno-economic parameters of its components. As output, it provides information for the design and operation of a minimum-cost energy system. As the ESM incorporates all of these data, it simultaneously serves as the software’s input and output data model.

As noted in the general schematic in Figure 1, the ETHOS.FINE interface offers the possibility of reading the input data from files or having them transferred by preceding software. However, in the second case, the information can be gathered step by step in the data model classes; for file-based information transfer, a single complex file containing all parameters must be provided. Both Excel and NetCDF file formats are accepted for this purpose. On the output side of the interface, the result data can also be saved in Excel or NetCDF form or visually depicted using a range of plotting functions.

The ETHOS.FINE interfaces for reading Excel and NetCDF files are implemented by one function each, which mainly obtain a path to the respective input file. Here, DataDesc offers the possibility, in addition to the superficial description of the string variables, of going in-depth and also describing the necessary internal structures of the input files (cf. Figure 4, lines 40–50). Thus, for the NetCDF interface, the control parameters and input variables were documented in the clearly structured hierarchical data format, which arranges the information according to entity types and, in each case, lists their attributes in accordance with their different dimensional characteristics. The documentation of the Excel data structure required more effort, as it does not group the data by entities but distributes them to different spreadsheets depending on their dimensional resolutions. The resulting tables, in which the multi-dimensional attribute characteristics of different entities are mapped by means of different index columns, required precise documentation to define the boundaries between individual datasets. In both cases, documentation could be created to help users understand the given file structures and arrange their own input data accordingly. The documentation effort depended on the straightness and non-ambiguity of the data model structures.

For the documentation of the programmatic interface, the constructors of the ESM and the component classes were described using DataDesc. Here, the use of Python’s typing library—and specifically the Annotated package^70^—enabled the inclusion of additional metadata into the code. Primitive variable names, comments, types, roles, and default values were easily mapped to the DataDesc annotation syntax (lines 8 ff) using generic type hints. Custom types, such as Panda’s dataframes, generally profit from more detailed structural information, which were integrated by adding metadata in form of key-value pairs to the annotation Annotated[T, x], where T is a given type and x any metadata. In addition, the value range constraints that some variables are subject to were integrated into the code using the Annotated package and later parsed into the documentation with the metadata elements contained in the DataDesc schema (line 25). Furthermore, complex data structures of ETHOS.FINE, as well as variables that are constrained to a permitted set of values, were described in detail (lines 13–20 and 30–32). To minimize the documentation effort, value sets and data structures that apply to several variables were documented once and then referenced repeatedly (line 37).

## Discussion

The FAIR principles and their adaptations to research software have received much attention and support. To effectively reuse a software, the software itself and its interfaces must be clearly defined and made understandable, ideally in a machine-actionable manner. However, most research software today is not documented or published in a way that provides detailed and machine-actionable interface descriptions. Instead, software metadata are often focused on the compact provision of general information, whereas documentation pages, including detailed, technical information, are primarily in natural language and not machine actionable.

Therefore, the DataDesc framework was presented in this article as an approach to describing the data models of software interfaces using detailed and machine-actionable metadata and as a FAIR advancement of existing research software metadata. In pointing out that there must be a differentiation between data structures and data formats, it was shown how to consistently describe data structures and, by doing this, support the interoperability of software to other programs and data files. In addition to a specialized metadata schema, an exchange format and support tools for the easy collection and automated publishing of software documentation were introduced. Using the ETHOS.FINE framework as an example, the practical applicability of DataDesc and its limitations were shown. It is hoped that DataDesc will facilitate the description of software interfaces with detailed and machine-actionable metadata enough to make it common practice, leading to increased interoperability, findability, and, therefore, reusability of research software.

### Limitations

The DataDesc schema currently has limitations in terms of capturing content dependencies of parameter values and structures. So, if the permitted value range, structure, or format of a variable depends on a user-specified value in another variable, then this cannot yet be mapped formally and in a machine-actionable format. Also, the description of procedural dependencies, in which the value of a variable influences the software-internal calculation processes, must be represented so far as free-text docstrings. An example from the application case for this is the component class that contains the Boolean parameter hasCapacityVariable, which, if set to true, causes the capacityVariableDomain and capacityPerPlantUnit variables to be ignored in the calculations. Work is currently underway to formally integrate this form of dependencies into the schema.

A similar form of content dependency type, which cannot currently be specified by DataDesc, can result from the interaction of different software components. In the application case shown, ETHOS.FINE integrates the tsam library for the purpose of temporal data aggregation and partially maps its external interface in its own interface. In a function of the ESM class, for example, the parameter aggregation method can be selected (lines 30–32). The permitted value set, which includes options like averaging or k-means, is specified by the external library and manually included in the ETHOS.FINE documentation. In the future, the documentation of independent programs could be integrated and reused automatically if they are also made machine actionable by means of the DataDesc schema.

In the area of utilities and publication pipelines, the DataDesc framework has limitations in that only a parser for Python-based research software and interfaces to five software platforms have been implemented to date. As part of the dissemination of the DataDesc framework, the aim is to encourage the community to support the coverage of additional programming languages and publication platforms.

### Outlook

Currently, the DataDesc framework is being incorporated into the Research Software Metadata Working Group of the Metadata Section of the National Research Data Infrastructure (NFDI) initiative to establish and support a comprehensive metadata vocabulary for research software and to aid the NFDI consortia in applying and extending the vocabulary according to their needs.^77^ Furthermore, DataDesc is currently being enhanced and applied to the description of datasets. With both software interfaces and data being described with DataDesc, upcoming research will lay the foundation for automatically composing and executing computational workflows. This will enable insights into how and with which data and programs software can be used and further increase the reuse and integration of research software and the reproducibility of computational research in the future.

## Resource availability

### Lead contact

Further information and requests for resources on the DataDesc framework should be directed to and will be fulfilled by the lead contact, Patrick Kuckertz (p.kuckertz@fz-juelich.de).

### Materials availability

This study did not generate new unique reagents.

### Data and code availability

The DataDesc metadata schema, the source code of the developed DataDesc utilities, and related example and documentation files are publicly available under the open MIT license at GitHub: https://github.com/FZJ-IEK3-VSA/DataDesc.^14^ In addition, these resources have been made available in their current version, v.1.0, under the CC0 public domain dedication at JülichDATA: https://doi.org/10.26165/JUELICH-DATA/DLCYV5.^15^

## Acknowledgments

The authors would like to thank the Federal Ministry for Economic Affairs and Energy of Germany (BMWi) for supporting this work with a grant for the project LOD-GEOSS (03EI1005B). Furthermore, the authors are grateful to the German federal government, the German state governments, and the Joint Science Conference (GWK) for their funding and support as part of the NFDI4Ing and the NFDI4Energy consortia, managed by the 10.13039/501100001659German Research Foundation (DFG) – 442146713 and 501865131. In addition, the work was supported by the Lower Saxony Ministry of Science and Culture within the Lower Saxony “Vorab” of the 10.13039/501100001663Volkswagen Foundation under grant 11-76251-13-3/19–ZN3488 (ZLE) and by the Center for Digital Innovation (ZDIN). This work was also supported by the 10.13039/501100001656Helmholtz Association as part of the program “Energy System Design.”

## Author contributions

Conceptualization, P.K., J.G., O.K., and S.F.; methodology, P.K., J.G., O.K., S.F., D.N., J.S., R.P., and F.E.; software, D.N., J.S., and O.K.; validation, R.P. and P.K.; investigation, P.K., J.G., O.K., D.N., J.S., R.P., and S.F.; data curation, J.S.; writing – original draft, P.K., J.G., O.K., D.N., J.S., R.P., and S.F.; writing - review & editing, P.K., J.G., O.K., D.N., J.S., R.P., S.F., F.E., N.P., J.M.W., A.N., S.A., and D.S.; visualization, P.K. and J.G.; supervision, D.S., S.A., and A.N.; project administration, P.K. and O.K.; funding acquisition, D.S., S.A., and A.N.

## Declaration of interests

The authors declare no competing interests.
